# Information for onchocerciasis control

**Published:** 2010-12

**Authors:** Adrian Hopkins

**Affiliations:** Director, Mectizan Donation Program, 325 Swanton Way, Decatur, GA 30030, USA. **www.mectizan.org**

**Figure:**
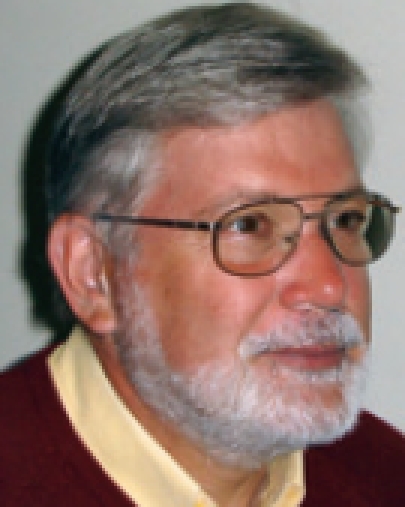


## Onchocerciasis and Mectizan

Mectizan® has been donated for the control of onchocerciasis for over twenty years, and also for the elimination of lymphatic filariasis for the last ten years. Merck & Co. Inc, or MSD as the company is known in many other parts of the world, are donating Mectizan® to as many who need it for as long as it is needed. But how much is needed? Individual patients with skin disease or eye disease need only small amounts, but where the prevalence of skin disease or eye disease in an area is high it is important to treat all eligible people in the community. If we treat around 65% of the population on a regular basis, the effects of the disease will be drastically reduced. If however we maintain 80% coverage of the total population (or 95-100% of the eligible population) onchocerciasis may eventually be eliminated. Mectizan® is usually given annually. In some areas, where it is feasible, treatment takes place twice and even four times a year to break the transmission cycle more quickly. That amounts to a lot of tablets!

## What information is needed?

In order to supply the correct amount of Mectizan®, we need data on the total population and/or the eligible population planned for treatment, the expected coverage level, and the planned number of treatment rounds during the year. The amount of Mectizan® needed can then be calculated. However, if the treatment planned was not completed the previous year, the amount has to be adjusted in order to avoid accumulating potentially expired drugs. It takes time to manufacture and ship drugs, and we need to know how much to produce in order to ensure that enough Mectizan® will be available when required. Prompt reporting of treatment, or even of difficulties in distribution, help to resolve some of these problems before they negatively impact drug delivery.

**Figure:**
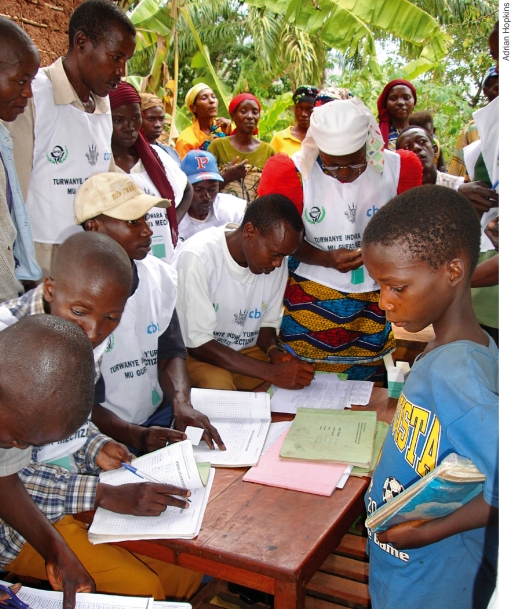
Community distributors are responsible for the collection of local treatment data. BURUNDI

Adrian Hopkins

**‘It takes time to manufacture and ship drugs, and we need to know how much to produce’**

The programme's major partner for distribution is the African Programme for Onchocerciasis Control (APOC). APOC has developed the strategy of community-directed treatment with ivermectin (CDTI). The communities, once sensitised, take ownership of the programme and run it themselves, including choosing community distributors and organising community supervision. For this to work, there has to be a close relationship with the most peripheral elements of the primary health care system, usually the health centre staff. We therefore also need information about how staff involved in onchocerciasis control are trained and functioning at all levels of the health service, and how many people need to be trained or retrained. This information is vital for planning and budgetary purposes, especially if other interventions are needed or if strategies change.

## How is the information collected?

Information is collected at different levels. At the community level, the community distributors are responsible for the collection of local treatment information, which is collected and collated (summarised) by the health centre nurse for the whole health area (the area supervised by a health centre). The nurse will add data on training, tablet inventory, etc. and send a report to the health district. The health district will add data on training, supervision, and other activities at the district level.

Normally, data is further centralised at the provincial level before being passed to the central level where it is forwarded to the supporting programmes and the drug donation programme. There has been an effort to get treatment data integrated into existing health management information systems in countries, but this has been a slow process, and there are many discussions about which indicators to use. As health systems are often weak at the periphery (e.g. remote or rural areas), community data may be collected by a supporting non-governmental organisation (NGO) in a parallel way and then forwarded to the government for their reports. Mectizan® is often donated through NGOs, so they are an important part of the process.

People need to be trained at the respective levels in order to collect this vital information. Once chosen by their community, distributors are trained in a very practical way, as close as possible to their community. Treatment is usually written down for each individual, and by family, in a locally bought exercise book or in specially printed registers. In some remote communities, volunteers may be illiterate. However, even in these circumstances, volunteers can be trained to use a simple tally sheet, which is often used to summarise data in any case. Training at the community level is usually done by the health centre staff who are in turn trained by staff at the district level, who have received their training at a provincial level. This form of ‘cascade’ training is a very effective process, but care must be taken to make sure the essential messages are relayed correctly at the relevant levels. Close supervision is required as incorrect data leads to incorrect tablet data or information for planning.

During the distribution process, the distributors are often helped by other volunteers to enter data into the exercise book or register. Having one page per household makes it easier to locate individuals for follow-up of the annual treatment or to find people who were absent at the time of treatment.

**Figure:**
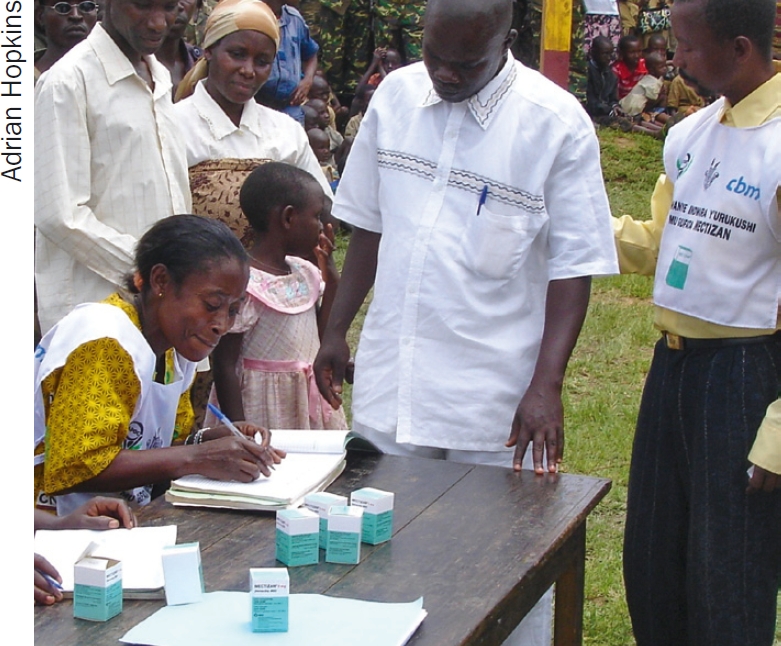
Community distributors can use the data they collect to do a follow-up. BURUNDI

Adrian Hopkins

The treatment summaries are prepared at the community level. These show numbers treated by gender and sometimes by dose (1-4 tablets) and also reasons for not taking the tablets (too young, pregnant, too ill, absent from the village, etc.). The health area nurse then collects data from all the communities and forwards it to the health district where it is centralised, sometimes computerised, and then forwarded to higher levels with their own activity report. Once again, NGOs sometimes facilitate this process.

## How is the information analysed and used?

The data collected by the volunteers are usually analysed at the community level and the following are calculated: total number of people treated, number of tablets used, and sometimes coverage (percentage or proportion of population treated). Volunteers may also use the family treatment sheets to follow up on those who had not received treatment, often revisiting their homes. They may also try to follow up on people refusing treatment. Specially trained community volunteers may also participate in this analysis and may calculate the coverage. Health centre nurses usually discuss the results with community volunteers and will check the coverage levels. At the district level, health centres are compared and the results are tabulated and coverage calculated before the report is forwarded to the provincial or central level.

The information is used in different ways at different levels:

- At the community level, the details of treatment are shared and the coverage is discussed with the community distributors, including the importance of high coverage for control or elimination. Problems or low coverage are discussed to try and resolve challenges. Sometimes, coverage is compared between communities to see “who is doing best.”
- At the health centre level, coverage is calculated: people responsible for high coverage are congratulated; discussions are held with those with low coverage and solutions examined. At this time, strategies for the next treatment round will be discussed and the needs for tablets will be calculated.
- At the district level, coverage is again the main issue as well as planning and budgeting for the next treatment round (training, retraining, further health education, etc.).
- At the central (national) level, the reports are used to calculate the next year's tablet request.

